# Household Processing Methods and Their Impact on Bioactive Compounds and Antioxidant Activities of Sweetpotato Genotypes of Varying Storage Root Flesh Colours

**DOI:** 10.3390/antiox11101867

**Published:** 2022-09-21

**Authors:** Flora C. Amagloh, Archileo N. Kaaya, Gaston A. Tumuhimbise, Arnold Katungisa, Francis K. Amagloh, Benard Yada

**Affiliations:** 1 Department of Food Technology and Nutrition, School of Food Technology, Nutrition and Bio-Engineering, College of Agricultural and Environmental Sciences, Makerere University, Kampala P.O. Box 7062, Uganda; 2 CSIR—Savanna Agricultural Research Institute, Tamale P.O. Box TL 52, Ghana; 3 Root Crops Program, National Crops Resources Research Institute, National Agricultural Research Organisation, Kampala P.O. Box 7084, Uganda; 4 Department of Food Science and Technology, Faculty of Agriculture, Food and Consumer Sciences, University for Development Studies, Tamale P.O. Box TL 1882, Ghana

**Keywords:** sweetpotato, functional food, bioactive compounds, phytochemicals, antioxidant activity, thermal processing, cooking, sub-Saharan Africa, Uganda, noncommunicable diseases

## Abstract

Sweetpotato storage roots, peeled and unpeeled, of varying flesh colours (white, cream, yellow, pale orange, deep orange, and purple) were spectrophotometrically evaluated for their bioactive compounds and antioxidant activities. Roots were boiled, steamed, baked, fried, or microwaved. The unpeeled roots had relatively higher (*p* < 0.001) bioactive compounds and antioxidant activities than the peeled ones. All cooking methods increased phenolic compounds, flavonoids, and tannins in all genotypes. Significant losses of total carotenoids occurred with all cooking methods (ranging from 24.18 to 172.76 µg/g in raw sweetpotatoes vs. 10.06 to 118.17 µg/g in cooked ones; *p* < 0.001), except the deep-orange-fleshed genotype, in which frying slightly increased carotenoids from 269.81 to 304.74 µg/g. Microwaving retained 69% vitamin C in the cream-fleshed one, the highest among the cooking methods. Anthocyanins decreased with baking and frying in the purple-fleshed one but increased with other methods; microwaving being highest at 13.9% (17.43 mg/g). While the 2,2′-azino-bis-3-ethylbenzthiazoline-6-sulphonic acid antioxidant activity decreased with all cooking techniques in some genotypes, ferricyanide-reducing antioxidant potential increased. The retention of bioactive compounds in sweetpotato storage roots depends on the processing method. Thus, to obtain the most health benefits, consumers should use different cooking methods but retain the peels.

## 1. Introduction

Sweetpotato (*Ipomoea batatas* (L.) Lam, Convolvulaceae) is a versatile root crop with various uses globally, both industrially and at the household level. However, recent research has focused on sweetpotato storage roots as a functional food due to the substantial amounts of bioactive compounds [1]. These bioactive compounds are mainly phytochemicals, and different sweetpotato genotypes contain varying compositions of polyphenols, flavonoids, stilbenes, lignans, glycolipids, carotenoids, anthocyanins, tocopherols, saponins, alkaloids, tannins, and terpenoids [1]. In addition, sweetpotato storage roots contain a variety of amino acids, vitamins (especially vitamin C, B_6_, and folate) and minerals, including manganese, copper, potassium, and iron [2].

According to the World Health Organization (WHO), low- and middle-income countries have prevalences of noncommunicable diseases (NCDs) that are disproportionately higher than in high-income regions of the world [3]. In sub-Saharan Africa (SSA), increasing urbanisation has led to unhealthier food choices and sedentary lifestyles. These factors have contributed to rising incidences of NCDs projected to be the primary cause of mortality by the year 2030 [4]. Consuming functional foods, such as sweetpotato, could be a food-based approach to aid in the management of NCDs in SSA [5] as it is cheap and easy to cultivate and aptly suited to the growing conditions of most ecologies in SSA [6].

Sweetpotato bioactive compounds have been reported to potentially exhibit health-promotion properties [2]. For example, anthocyanins from purple-fleshed sweetpotato (PFSP) have been shown to have higher antioxidant and anticancer properties when compared with other highly pigmented foods, such as aubergines, plums, and red onions. This is because PFSP anthocyanins mainly occur in acylated complexes with phenolic compounds, thereby making them more stable [7]. Further, phenolic compounds have antioxidant properties; they inhibit cancer cell growth and aid in managing chronic diseases, such as type 2 diabetes [8].

Sweetpotato storage roots vary in skin and flesh colour [9]. However, the white- and cream-fleshed ones are predominant in most SSA countries, including Uganda [10]. During the last decade, orange-fleshed sweetpotato (OFSP) has gained popularity in SSA, mainly as a dietary source of β-carotene to alleviate vitamin A deficiency [11]. On the other hand, PFSP genotypes have not received much attention in SSA, and Uganda is at an advanced stage for varietal release [12].

There is scanty information on profiling sweetpotato as a functional food in SSA, particularly when processed for consumption [13,14]. The inherent bioactive compounds in white-, cream-, yellow-, pale-orange-, deep-orange-, and purple-fleshed uncooked sweetpotato storage roots have recently been evaluated [15]. However, as these roots are not consumed raw, there is a need to determine how standard domestic processing methods influence the various bioactive compounds’ content, and consequently, the antioxidant activities.

Earlier investigations have indicated that bioactive compounds undergo changes during thermal treatment, and these changes are determined by the process temperature, time, and presence of water or steam [16,17]. Anthocyanins, highly reactive molecules, may be degraded due to changes in pH, temperature, oxygen, enzymes, or their structure and concentration [18]. The effects of cooking on sweetpotato anthocyanins have yielded mixed results in different studies. An increase in anthocyanins was reported with boiling, steaming, baking, and microwaving [19], and a decrease was observed with boiling, steaming, roasting, and baking [20,21]. In another study on deeply fleshed purple sweetpotato, an increase in anthocyanins occurred after boiling, a decrease occurred after baking and frying, and steaming and microwaving had no significant effect on the level [22]. These conflicting data may be due to varietal effects and the type of anthocyanins present, environmental conditions for growing storage roots, or analytical procedures used, as earlier reported by [19,23].

Due to the importance of vitamin C in nutrition, such as its role in collagen synthesis, wound healing, protein metabolism, and immune function [24], investigation of an optimal cooking method that minimises losses is warranted. Regarding phenolic compounds, some researchers have reported an increase after baking or roasting of sweetpotato roots [19] and a decrease with steaming and flour production [25].

Carotenoids in sweetpotato roots were reported to decrease with boiling, steaming, and roasting [20,25] but increase when fried [14]. Based on the results of these findings, different processing methods could impact sweetpotato bioactive compounds differently. Against this background, therefore, it is expedient to evaluate locally grown sweetpotato genotypes. To ascertain the suitability of the different storage root flesh colours of sweetpotato grown in Uganda as a functional food, they have to be subjected to thermal processing methods, as would be performed in most households, and their effect on the bioactive compounds’ concentrations would have to be measured. Traditional cooking of sweetpotato storage roots in SSA includes boiling, steaming, frying, roasting, and drying [26].

This study investigated the changes in total phenolics, vitamin C, flavonoids, carotenoids, anthocyanins, saponins, alkaloids, tannins, and antioxidant activities of six (6) sweetpotato genotypes of varying flesh colours grown in Uganda after applying different processing methods.

## 2. Materials and Methods

### 2.1. Experimental Design and Sample Preparation

The study employed a 2 × 6 × 6 factorial experimental design: two (2) peel treatments (peeled and unpeeled), six (6) sweetpotato genotypes, and six (6) cooking methods; uncooked storage roots were included as a control sample.

The sweetpotato genotypes were: a white-fleshed landrace (‘Ssetyabule’), a cream-fleshed variety (NASPOT 11), a pale-yellow-fleshed variety (NAROSPOT 1), a pale orange-fleshed variety (NASPOT 8), a deep-orange-fleshed variety (NASPOT 13 O), and a deep-purple-fleshed advanced yield trial clone (PF-167) [27,28,29]. The cooking methods employed were boiling, steaming, baking, frying, and microwaving.

The detailed methods for sample preparation have been outlined in a previous publication [15]. Briefly, sweetpotato samples were harvested between 4 and 5 months after planting. Harvesting was separately performed thrice, resulting in triplicate independent biological samples. These replicates were used for all cooking methods and laboratory analyses. Different sizes of roots were randomly selected for each genotype. The roots were washed with tap water to remove debris. After air-drying, roots were stored at room temperature for three (3) days before processing. For processing, roots were longitudinally divided into two (2) equal parts. For one half, the peel was completely removed with a kitchen knife and designated as the peeled samples. The other half of each root was left unpeeled and designated as the unpeeled samples.

### 2.2. Cooking Methods

The various cooking methods applied to the different sweetpotato storage roots are summarised in Table 1. These methods were used for both peeled and unpeeled samples. Initial experiments were conducted to determine the cooking time required for each method. Roots were deemed cooked when a stainless-steel rod easily penetrated the flesh while applying reasonable pressure.

All the cooked samples were allowed to cool to room temperature, packaged into polyethylene bags, kept in a freezer (−18 to −20 °C) for at least a week, and subsequently lyophilised for 72 h. Raw storage roots, used as the control treatment, were cut into 1.5 mm thick slices with a mechanical slicer and frozen straight away.

All samples, except fried, were finely pulverised with an electric mill to pass a 0.42 mm aperture sieve. The fried samples were milled with a Kenwood^®^ grinder, sieved with a 1 mm mesh, and sieved with a 0.5 mm mesh afterwards. After milling, all samples were kept in polyethylene bags and stored at −18 to −20 °C prior to analysis.

### 2.3. Preparation of Sample Extract

For the extraction of total phenolic compounds, total flavonoid content, total alkaloid content, total tannin content, and antioxidant activity, a sample extract was prepared following the method recommended by Ooi et al. [30]. Twenty millilitres of freshly prepared 80% acetone was added to 5 g of freeze-dried sweetpotato flour. Acetone was used because of its ability to dissolve both polar and nonpolar compounds [30]. The suspension was homogenised with a vortex shaker (Labnet International Inc., Edison, NJ, USA) for 1 min and then for 1 h on an orbital shaker (Heidolph UNIMAX 1010 DT, Schwabach, Germany). Samples were then centrifuged (HERMLE Benchmark Centrifuge, Z 326 K, Gosheim, Germany) at 6000 rpm at 4 °C for 10 min. The supernatant obtained was used as an extract for the analysis. For total saponins, however, ethanol was used in the extraction as acetone has been reported to interfere with colour formation, resulting in falsely increasing saponin content [31]. Anthocyanins were extracted with a methanol–water mixture, as recommended by Giusti and Wrolstad, to give an accurate measurement even in the presence of other interfering compounds [32].

### 2.4. Assay of Bioactive Compounds and Antioxidant Activities

The analytical methods for the determination of bioactive compounds and antioxidant activities have been previously published [15].

### 2.5. Statistical Analysis and Data Presentation

The ‘Agricolae’ Package in the R Statistical Programme (v 4.1.0) was employed for multifactor analysis of variance (ANOVA) to compare the treatment effects of sweetpotato genotype, peel condition, and cooking method on the response variables. Post hoc test (Fisher’s LSD method) was applied for multiple mean comparisons when *p* < 0.05. Values were reported as means ± standard deviations (SD), on a dry weight basis.

In this study, although the third-level interactions among genotype, cooking method, and peel condition were significant, to avoid complexity, the results of the second-level interactions or main effects, where significant, have been presented. The interaction effects between sweetpotato genotype and cooking method and the main effect of peeling have been presented. Cooking method vs. peel condition (Table S1, Figure S1) and genotype vs. peel condition (Table S2, Figure S2) have been presented as supplementary attachment to this manuscript. Pearson’s correlation analysis was carried out between the response variables. Associations among response variables were determined by principal component analysis (PCA).

## 3. Results and Discussion

### 3.1. Bioactive Compound Content as Influenced by Genotype and Cooking Method

The results of the effects of the various cooking methods on the total phenolic, flavonoid, carotenoid, anthocyanin, vitamin C, alkaloid, saponin, and tannin contents of all the sweetpotato genotypes are presented in Table 2. Sweetpotato storage roots differed significantly (*p* < 0.001) by genotype and cooking method for all the phytochemicals analysed.

#### 3.1.1. Total Phenolic Compounds and Total Flavonoid Content

Generally, the total phenolic compounds and flavonoids significantly (*p* < 0.05) increased in all genotypes, irrespective of the cooking method applied (Table 2). The total phenolic content ranged between 4.95 mg GAE/g in raw NASPOT 8 and 190.96 mg GAE/g in baked ‘Ssetyabule’. Compared with the control, cooking increased the total phenolic compounds by 186–211% in ‘Ssetyabule’, 111–149% in NASPOT 11, 104–362% in NAROSPOT 1, 115–243% in NASPOT 8, 131–331% in NASPOT 13 O, and 139–163% in PF-167.

Bound phenolics were released from ruptured cell walls when heating was applied, accounting for the increase in total phenolic compounds in the cooked samples [33]. In addition, the degradation of polyphenol oxidase at temperatures greater than 70 °C could have accounted for this observation. This enzyme is responsible for catalysing the oxidation of phenolic compounds into quinones that bind to amino acids. Thus, if it is degraded during cooking, intact phenolic compounds are available for extraction during analysis [34]. The authors of a review article opined that those studies in which phenolics were lost during boiling could be due to the water-soluble phenolic compounds leaching into the water or changes in the specific food matrix involved [35]. However, in this study, there was no excess water remaining after boiling.

In all the sweetpotato genotypes, baking resulted in the highest content of phenolic compounds, increasing from about 1.5-fold in NASPOT 11 (134.59 mg GAE/g vs. 90.18 mg GAE/g in the raw roots) to 3.3-fold in NASPOT 13 O (18.86 mg GAE/g vs. 5.69 mg GAE/g in the raw roots). The combination of high temperature with long time during baking may have facilitated the cleavage of more phenol–sugar glycosidic bonds, releasing phenolic aglycones, as reported earlier [13]. The baking method and the white-fleshed sweetpotato (WFSP) had a significantly higher phenolic content (190.96 mg GAE/g; *p* < 0.001) than the other genotypes, and could thus play a significant role in the management of NCDs, such as type 2 diabetes [36].

#### 3.1.2. Total Carotenoid Content

In all sweetpotato genotypes studied, except NASPOT 13 O, cooking generally decreased total carotenoids, ranging from 3.4% reduction in fried NAROSPOT 1 to 59.9% in baked NASPOT 11 (Table 2). The most significant reduction observed in the baking method was expected, as has been reported elsewhere that carotenoids are extensively degraded with higher temperatures and longer processing times [17].

Nevertheless, in NASPOT 13 O, the deep-orange-fleshed variety with the highest content of total carotenoids (269.81 µg/g), frying significantly increased total carotenoids by 12.9% (304.74 µg/g; *p* < 0.001), while steaming had no significant effect, retaining about 99.2% of carotenoids in the raw sample (267.64 µg/g; *p* > 0.001). Similar results were reported by Abong’ et al. [14], in which β-carotene significantly increased in fried OFSP storage roots by 68% to 111%. With the lipophilic nature of carotenoids, the addition of oil significantly enhances micellarisation, where the molecules are solubilised in the oil droplets in the food matrix, increasing their availability during analysis [26].

Although all cooking methods decreased carotenoids in the other genotypes, frying resulted in the highest retention. Percentages of carotenoid retention during frying were 55.1% in ‘Ssetyabule’, 84.7% in NASPOT 11, 96.6% in NAROSPOT 1, 88.5% in NASPOT 8, and 88.3% in PF-167. Bechoff and coauthors, in their study on the retention of β-carotene in foods containing OFSP flour, observed up to 97% β-carotene retention in some samples cooked with oil [26]. Compared with other cooking methods, frying can be recommended when processing OFSP genotypes if one is interested in increasing the dietary intake of provitamin A.

#### 3.1.3. Total Monomeric Anthocyanin Content

Apart from PFSP, anthocyanin content generally decreased after cooking in all the genotypes, although most were not significantly different from the content of the raw samples. The anthocyanin content remaining after cooking in the other genotypes ranged from 0.40 mg/g in baked NASPOT 11 to 2.43 mg/g in microwaved NAROSPOT 1. However, in PF-167, boiling, steaming, and microwaving significantly (*p* < 0.001) increased anthocyanins by 12.0% (17.13 mg/g), 9.0% (16.67 mg/g), and 13.9% (17.43 mg/g), respectively. On the contrary, baking and frying significantly (*p* < 0.001) reduced their content by 19.9% and 8.9%, respectively (12.23 mg/g and 13.92 mg/g vs. 15.29 mg/g in the control) (Table 2). Baking and frying temperatures were higher than boiling, steaming, and microwaving (Table 1) and, thus, may have resulted in greater degradation of anthocyanins, as in this study. In addition, baking exposed samples to a higher temperature for a longer time, thus reducing anthocyanins more than in frying. This finding resonates with a study in which boiling increased anthocyanins by 6.6% but baking and deep frying decreased it by 32.0% and 11.0%, respectively [22]. This was attributed to different types of cooking having different effects on specific anthocyanin monomers and causing them to behave differently [22]. Thus, microwaving and PF-167 could be recommended when considering anthocyanin content in the cooked sweetpotato genotypes.

PF-167 may have behaved differently than the other genotypes used in this study because in the other genotypes, the anthocyanin pigments occurred mainly in the skin, but not the flesh, compared with PF-167, in which both the flesh and the skin had abundant anthocyanins [15]. Although the individual anthocyanin monomers were not elucidated in this study, different anthocyanins may likely be present in the skin vs. the flesh of sweetpotatoes.

#### 3.1.4. Vitamin C Content

All cooking methods significantly decreased (*p* < 0.001) vitamin C content in all the sweetpotato genotypes. Vitamin C content in the raw storage roots ranged between 35.65 µg AAE/g in ‘Ssetyabule’ and 140.05 µg AAE/g in NASPOT 11, and in the cooked samples, from 8.33 µg AAE/g in baked NASPOT 11 to 91.91 µg AAE/g in microwaved NASPOT 8 (Table 2). This reduction was expected because vitamin C is highly susceptible to oxidation, especially at higher temperatures [14]. However, microwaving was the most favourable for all genotypes, retaining up to 68.7% in NASPOT 8.

The most significant loss of vitamin C occurred in NASPOT 11, ranging from a retention of 5.9% (8.33 µg AAE/g) in baking to 18.7% (26.21 µg AAE/g) in microwaving compared with 140.05 µg AAE/g in the uncooked storage roots. Similarly, during cooking of potato tubers in a previous study, microwaving proved to be the most favourable method, retaining up to 90.1% vitamin C content in their samples, whereas grilling, the most destructive method, retained 78.5% [37]. Another study comparing baking temperature and time effects on vitamin C retention in sweetpotato suggested that cooking time may be more important than temperature in determining vitamin C losses [38]. Thus, microwaving, but not baking, may be a suitable method for preserving vitamin C content in sweetpotato. For our study, microwaving NASPOT 8 yielded the highest vitamin C content (91.91 µg AAE/g; *p* < 0.001) among all cooked samples.

#### 3.1.5. Total Alkaloid, Saponin, and Tannin Contents

All cooking methods, except baking, significantly reduced (*p* < 0.001) total alkaloid content independent of sweetpotato genotype, ranging from 22.22 µg CE/g in boiled NAROSPOT 1 to 157.77 µg CE/g in baked ‘Ssetyabule’ (Table 2). In all genotypes, boiling resulted in the most significant decrease in alkaloid content. Baking, although it led to slight increases in alkaloid content, had no significant effect (*p* > 0.001) compared with the raw samples. These results are consistent with a study in which boiling of potato tubers decreased alkaloids the most compared with baking and microwaving [39]. Baking and ‘Ssetyabule’ were the most favourable cooking method and genotype with the highest alkaloid content. Although alkaloids, especially glycoalkaloids, were historically regarded as toxic compounds, more recent research has considered these compounds safe in doses not exceeding 3–10 mg/g. Below these levels, alkaloids are known to be potentially beneficial, especially in preventing or managing type 2 diabetes [40]. This antidiabetic property of alkaloids is potentially achieved through reducing diabetic-induced oxidative stress, inhibition of carbohydrate digesting enzymes by competing for binding sites, and regeneration of pancreatic cells and, therefore, insulin secretion [41,42].

Cooking generally increased total saponins in all the genotypes (Table 2), ranging from 161.29 mg AE/g in fried NAROSPOT 1 to 491.18 mg AE/g in baked ‘Ssetyabule’. Apart from baked ‘Ssetyabule’ having significantly higher saponin content (*p* < 0.001), all the other cooking methods did not differ significantly in their effect on saponins in the other genotypes. This suggests that any thermal processing would likely enhance the content of saponins in sweetpotato. Previous literature has documented saponins as having antiviral activity against HIV, cholesterol-lowering activity, activity for the prevention of osteoporosis, and anticancer activity through the reduction of inflammation [43]. In addition, saponins could lower plasma glucose levels and offer protection against oxidative stress [44]. Therefore, saponin-rich foods, such as sweetpotato, could provide physiological benefits for people with such conditions.

Total tannin content increased significantly (*p* < 0.001) with all cooking methods in all the sweetpotato genotypes. Cooking PF-167 resulted in its total tannins being significantly higher (*p* < 0.001) than in all the other genotypes, although the tannin content in raw ‘Ssetyabule’ was higher than in raw PF-167 (2.68 mg TA/g vs. 1.68 mg TA/g, respectively). Microwaving increased total tannins, the most in PF-167 by 7.3-fold (12.23 mg TA/g), while frying resulted in the slightest increase of 5-fold (8.52 mg TA/g). Contrary to these results, total tannins decreased during the cooking of Kenyan OFSP storage roots [14], possibly due to the type of specific tannins present in those genotypes. Tannins are a class of diverse polymeric phenolic compounds abundant in tea, cocoa, wine, some fruits, vegetables, and grains [45]. Due to their varied physiological roles, they may be regarded as negative or positive constituents. Tannins may be considered antinutrients because of their ability to chelate metal ions and bind to dietary proteins and digestive enzymes in the gut [46]. On the other hand, they are potentially known to scavenge unhindered free radicals by binding to them and thus acting as antioxidants. They may also have antimicrobial, anti-inflammatory, and anticarcinogenic benefits [46]. From the results obtained in this study, microwaving was the most favourable cooking method for PF-167, the genotype that yielded the highest total tannin content (Table 2).

### 3.2. Bioactive Compound Content as Influenced by Peeling

Table 3 shows the results of the main effect of peeling on the content of the bioactive compounds. Whether in the raw or cooked state, peeling significantly decreased (*p* < 0.001) bioactive compound content. As expected, unpeeled samples contained higher bioactive compounds because these secondary metabolites are produced in response to environmental conditions, thus occurring in copious quantities in the skin than in the flesh [47].

### 3.3. Variations in Antioxidant Activities

**Figure 1:**
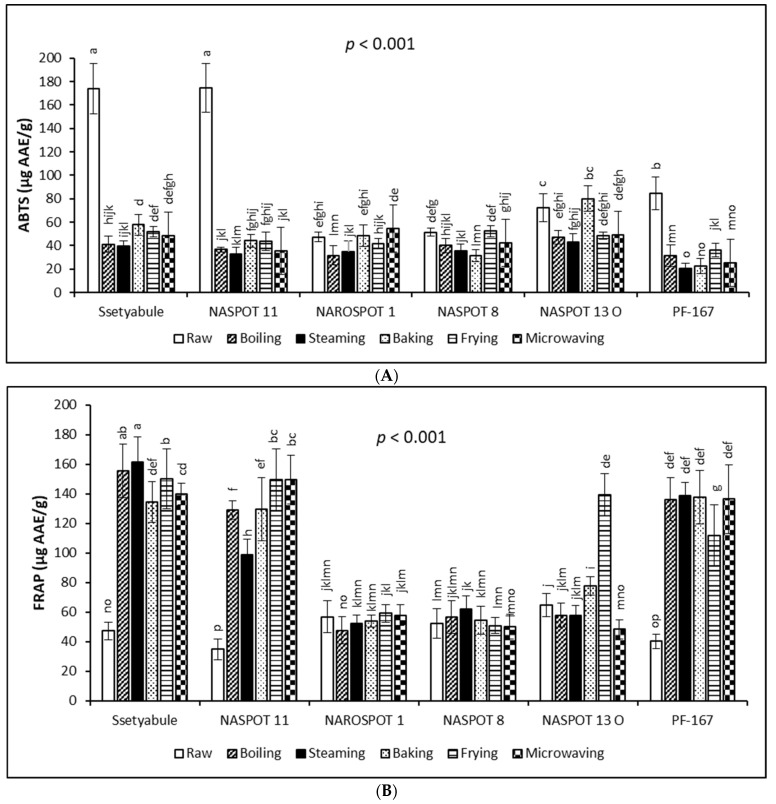
Changes in antioxidant activities of sweetpotato genotypes during cooking by ABTS assay (**A**) and by FRAP assay (**B**). Each bar represents the mean of three independent biological replicates (*n* = 3) with the error bar showing the standard deviation. Bars with different letters on top are significantly different at *p* < 0.001.

The antioxidant activities of the six (6) processed sweetpotato genotypes are shown in Figure 1. The ABTS antioxidant activity varied significantly (*p* < 0.001) with genotype and cooking method, ranging from 20.77 µg AAE/g in steamed PF-167 to 174.81 µg AAE/g in raw NASPOT 11 (Figure 1A). Significant variations (*p* < 0.001) also existed with genotype and cooking method for FRAP, ranging from 34.94 µg AAE/g in raw NASPOT-11 to 161.68 µg AAE/g in steamed ‘Ssetyabule’ (Figure 1B).

While ABTS decreased with all cooking methods in ‘Ssetyabule’, NASPOT 11, and PF-167, FRAP increased in these genotypes with cooking. However, in NAROSPOT 1, NASPOT 8, and NASPOT 13 O, both ABTS and FRAP obtained mixed results of either increase or decrease. Considering the cooked samples, ABTS was highest in baked NASPOT 13 O (80.04 µg AAE/g) and lowest in steamed PF-167 (20.77 µg AAE/g). FRAP was highest in steamed ‘Ssetyabule’ (161.68 µg AAE/g) and lowest in boiled NAROSPOT 1 (47.63 µg AAE/g).

These opposing results by the two assays are possibly because the radical scavenging antioxidant activity (as measured by ABTS) and the reducing potential antioxidant activity (as measured by FRAP) are based on different reaction mechanisms [48]. It has been suggested that the loss of antioxidant activity during the processing of fruits and vegetables may occur due to oxidation, enzymatic or nonenzymatic conversion, thermal degradation, and leaching [16]. Contrarily, increasing antioxidant activity during processing has been ascribed to softening of the food matrix during cooking, leading to a greater extractability of the antioxidant constituents, which further convert to more antioxidant compounds [49]. In addition, Tang et al. [20] suggested that different bioactive compounds might react differently to different antioxidant assays.

Although the FRAP values for raw ‘Ssetyabule’, NASPOT 11, and PF-167 were lower than the values for raw NAROSPOT 1, NASPOT 8, and NASPOT 13 O, cooking the former resulted in significantly higher FRAP values than the latter genotypes. These observed differences among the sweetpotato genotypes could be due to the interference of other compounds, such as ascorbic acid, carotenoids, and other oxidising agents and reducing sugars that are present in varying amounts in the different genotypes and react differently to thermal processing [13]. Further, fried NASPOT 13 O gave a significantly higher FRAP value (139.46 µg AAE/g; *p* < 0.001) than the other yellow- and orange-fleshed genotypes. The higher phenolic compound content in cooked ‘Ssetyabule’, NASPOT 11, and PF-167 and the highest total carotenoid content in fried NASPOT 13 O (Table 2) suggest that phenolic compounds and carotenoids may be potent antioxidants. Similar to these results, FRAP antioxidant activity increased in cooked sweetpotato [13], as well as in cooked carrots, courgettes, and broccoli [49] compared with their raw forms. For the total antioxidant activities observed in this study, baking and NASPOT 13 O favoured ABTS the most, while the highest FRAP was obtained with steamed ‘Ssetyabule’.

Antioxidant activities for the peeling treatment also varied significantly (*p* < 0.001) for both ABTS and FRAP. For both ABTS and FRAP, peeling significantly reduced antioxidant activities in the samples. For ABTS, the mean value for unpeeled samples (61.36 ± 30.65 µg AAE/g) was almost 1.5 times that for the peeled samples. For FRAP, the unpeeled roots (101.96 ± 46.96 µg AAE/g) recorded 1.3 times the value of the peeled ones. The higher content of phytochemicals in the skin versus the flesh of sweetpotato is reflected in these results [47].

### 3.4. Correlations between the Bioactive Compounds and Antioxidant Activities

**Figure 2:**
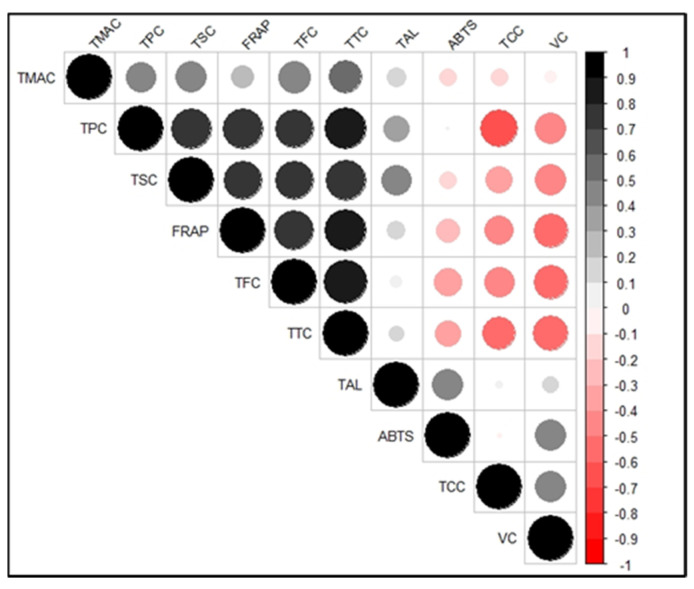
Correlation matrix showing the correlations between each pair of variables (bioactive compounds and antioxidant activities) of sweetpotato genotypes. An increasing shade of blackness depicts am increasing positive correlation. An increasing shade of redness depicts an increasing negative correlation. TMAC = total monomeric anthocyanin content; TPC = total phenolic compounds; TSC = total saponin content; TFC = total flavonoid content; TTC = total tannin content; TAL = total alkaloid content; TCC = total carotenoid content; VC = vitamin C; FRAP = ferricyanide-reducing antioxidant potential; ABTS = ABTS radical scavenging antioxidant potential.

The heat map in Figure 2 shows the correlations between the bioactive compounds and antioxidant activities of the sweetpotato genotypes studied. Significant positive correlations (*p* < 0.001) existed between the ABTS antioxidant activity and total alkaloids and vitamin C content while significant negative correlations were observed between ABTS and total tannin (*p* = 0.007) and flavonoid (*p* = 0.008) content. About 23% and 21% of the variation observed in ABTS antioxidant activity were associated with total alkaloids and vitamin C content, respectively. This relationship reflects the decrease in these two bioactive compounds during cooking (Table 2), mirrored by the reduction in ABTS with cooking (Figure 1A).

For the FRAP antioxidant activity, significant positive correlations (*p* < 0.001) occurred with total phenolics, flavonoids, saponins, and anthocyanins (*p* = 0.039). On the other hand, significant negative correlations existed with total carotenoids (*p* = 0.004) and vitamin C (*p* < 0.001). Variations in FRAP mainly were associated with total tannins (67%), phenolics (62%), flavonoids (56%), and saponins (52%). The association of these bioactive compounds with the FRAP antioxidant activity is reflected in their increase during cooking (Table 2), which was the same as FRAP (Figure 1B). Therefore, changes in these phytochemicals during the cooking of sweetpotato could be used as indicators in assessing FRAP antioxidant activity.

The strongest positive correlation was between total flavonoid and total tannin content (r = 0.90; *p* < 0.001), indicative of the fact that both compounds belong to the class of phenolic compounds [47] and may thus behave similarly. The strongest negative correlation was between total phenolic and total carotenoid content (r = −0.67; *p* < 0.001), consistent with the observation that the white-, cream-, and purple-fleshed sweetpotato genotypes had higher phenolics and lower carotenoids than the yellow- and orange-fleshed genotypes (Table 2).

### 3.5. Interrelationships among the Bioactive Compounds and Antioxidant Activities with PCA

PCA was used to summarise the relationships among all the parameters analysed in this study. The PCA yielded 10 principal components, out of which the first 3 had eigenvalues greater than 1 (PC1 = 5.23; PC2 = 1.76; PC3 = 1.05), cumulatively explaining 80.5% of the total variance.

**Figure 3:**
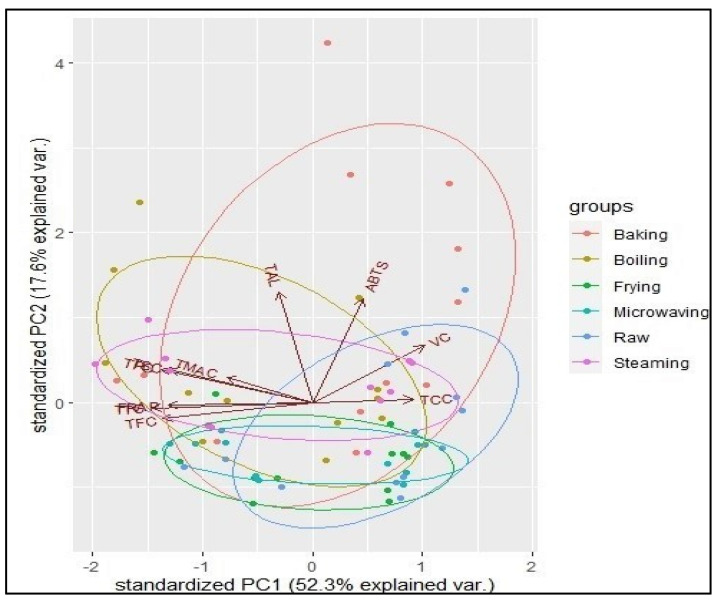
PCA biplot showing the interrelationships among the individual bioactive compounds and antioxidant activities of sweetpotato genotypes on the axes of principal components 1 (x-axis) and 2 (y-axis). The vectors represent the dependent variables of the dataset, and the dots represent the observations. TCC = total carotenoid content; VC = vitamin C content; ABTS = ABTS radical scavenging antioxidant potential; TAL = total alkaloid content; FRAP = ferricyanide-reducing antioxidant potential; TMAC = total monomeric anthocyanin content; TSC = total saponin content; TPC = total phenolic compounds; TTC = total tannin content; TFC = total flavonoid content.

Figure 3 shows the biplot of principal components 1 and 2 that cumulatively explained 69.9% of the total variation. Acute angles between total phenolics, flavonoids, anthocyanins, tannins, saponins, and FRAP antioxidant activity reflect the close relationship of those bioactive compounds with FRAP rather than ABTS. Additionally, the similar vector directions exhibited by these bioactive compounds indicate a strong relationship.

However, total alkaloid, carotenoid, and vitamin C contents were more closely associated with ABTS than FRAP, buttressing the observation of their reduced content after cooking, just like in ABTS. The opposite directions of the vectors for total phenolics, flavonoids, anthocyanins, tannins, and saponins on one hand and vitamin C and carotenoids illustrate an inverse relationship between these two classes of bioactive compounds and their behaviour during the cooking of the sweetpotato genotypes (i.e., increasing for the former and decreasing for the latter). These observations suggest that the more a genotype is rich in carotenoids, the higher is its vitamin C content, and the lower is its phenolic compound content.

As evidenced by the ellipses of the various cooking methods, baking, boiling, and steaming were more strongly associated with all the bioactive compounds and antioxidant activities evaluated, contributing to a more significant variation in the data. Frying and microwaving in opposition did not contribute to a large variance in the bioactive compound content or in the antioxidant activities.

## 4. Conclusions

The influence of domestic cooking methods on the bioactive compounds and antioxidant activities of sweetpotato genotypes of varying storage root flesh colours was studied. Sweetpotato genotype, cooking method, and peeling all had significant effects on the bioactive compounds analysed. In all genotypes, baking resulted in the highest levels of phenolic compounds. All cooking methods decreased total carotenoids in all genotypes, except in the deep-orange-fleshed genotype, in which total carotenoids increased slightly with frying. Significant losses of vitamin C occurred with all cooking methods, though for all genotypes, microwaving was the most favourable. Baking and frying degraded total anthocyanins in the PFSP, but microwaving increased them the most. Independent of the genotype, boiling decreased total alkaloids the most, while baking did not differ significantly from the raw. For different genotypes, different cooking methods affected their antioxidant activities differently due to the influence of a specific component content. All samples that were peeled before cooking had significantly lower bioactive compounds and antioxidant activities compared with their unpeeled forms. Thus, different household cooking methods applied to sweetpotato storage roots affect the bioactive compounds and antioxidant activities differently, but it is more advantageous to choose processing methods that can utilise the peels. Further studies are required to ascertain the current state or potential of the consumption of unpeeled cooked storage roots among households in Uganda.

## Figures and Tables

**Table 1:** Different cooking methods applied to sweetpotato storage roots.

Cooking Method	Process
Boiling	Water was brought to boil in a covered saucepan. The storage roots were diced into 2.5 cm^3^ portions with a manual all-purpose potato cutter (Jumbo Potato Cutter, JET-L-HPG-062, Jiangsu China), placed in the boiling water at 96–97 °C, and boiled for 25 min. The ratio of roots to water used was 2:1.
Steaming	Water was brought to boil in an electric rice cooker (Geepas-GRC 4331-3.2 L, Guangzhou, China). Diced sweetpotato storage roots (2.5 cm^3^ portions) were placed in a steam basket on top of boiling water, covered, and steamed at 93–95 °C for 30 min. The ratio of roots to water was 1:1.
Baking	Diced sweetpotato storage roots (2.5 cm^3^ portions) were single-layered in a preheated aluminium baking pan, covered with aluminium foil, and baked in an electric oven at 180 °C for 1 h.
Frying	Sweetpotato storage roots were sliced into approximately 1 cm thick chips with a kitchen knife. The chips were deep-fried with preheated unfortified sunflower oil at 160 °C for 8 min, using an electric deep fryer (Saachi-3 L, Shanghai, China). Chips were allowed to drain in a stainless-steel basket for 3 min after frying.
Microwaving	The 2.5 cm^3^ portions of diced sweetpotato storage roots were microwaved on medium high for 15 min, using a 700 W microwave (HiSense-H20-MOMMI, Qingdao, China).

**Table 2:** Bioactive compound content of sweetpotato storage roots as influenced by genotype and cooking method.

Genotype	Cooking Method	TPC (mg GAE/g)	TFC (mg QE/g)	TCC (µg/g)	TMAC (mg/g)	VC (µg AAE/g)	TAL (µg CE/g)	TSC (mg AE/g)	TTC (mg TA/g)
‘Ssetyabule’	Raw	90.35 ± 7.03 ^j^	1.19 ± 0.35 ^q^	24.18 ± 1.91 ^qr^	0.86 ± 0.02 ^fgh^	35.65 ± 9.91 ^ij^	153.53 ± 28.15 ^a^	287.68 ± 27.37 ^de^	2.68 ± 1.66 ^lmno^
	Boiling	167.73 ± 20.24 ^cd^	5.86 ± 1.45 ^fghi^	10.43 ± 3.24 ^t^	0.68 ± 0.17 ^fgh^	11.27 ± 3.59 ^mno^	52.81 ± 16.08 ^fghij^	276.46 ± 21.16 ^defg^	6.75 ± 0.97 ^ij^
	Steaming	173.23 ± 10.54 ^bc^	5.92 ± 1.43 ^fgh^	10.27 ± 2.76 ^t^	0.66 ± 0.15 ^fgh^	8.42 ± 2.26 ^o^	57.88 ± 15.04 ^fg^	422.68 ± 36.44 ^bc^	7.63 ± 1.37 ^fgh^
	Baking	190.96 ± 18.23 ^a^	5.69 ± 1.77 ^ghi^	11.44 ± 1.79 ^t^	0.48 ± 0.11 ^h^	10.44 ± 3.11 ^no^	157.77 ± 22.05 ^a^	491.18 ± 21.50 ^a^	7.40 ± 0.92 ^ghi^
	Frying	171.69 ± 16.66 ^bc^	6.14 ± 1.53 ^efgh^	13.33 ± 2.01 ^st^	0.46 ± 0.10 ^h^	13.46 ± 3.56 ^mno^	56.43 ± 19.41 ^fg^	439.96 ± 26.28 ^b^	6.59 ± 0.87 ^j^
	Microwaving	173.21 ± 12.13 ^bc^	6.29 ± 2.14 ^efg^	10.06 ±3.04 ^t^	0.74 ± 0.11 ^fgh^	18.65 ± 6.93 ^lm^	60.90 ± 10.23 ^ef^	409.01 ± 20.24 ^bc^	8.08 ± 1.32 ^ef^
NASPOT 11	Raw	90.18 ± 11.54 ^j^	1.07 ± 0.43 ^q^	35.57 ± 8.80 ^p^	1.24 ± 0.34 ^efgh^	140.05 ± 29.57 ^a^	54.90 ± 14.34 ^fg^	96.68 ± 19.40 ^o^	1.86 ± 0.02 ^pq^
	Boiling	130.12 ± 10.54 ^fg^	8.14 ± 1.44 ^c^	21.87 ± 4.79 ^qrs^	1.23 ± 0.31 ^efgh^	15.31 ± 4.58 ^mno^	32.38 ± 11.16 ^lmn^	220.51 ± 31.76 ^ijkl^	8.46 ± 0.44 ^e^
	Steaming	121.04 ± 18.25 ^gh^	6.41 ± 1.93 ^ef^	20.29 ± 5.82 ^rs^	1.18 ± 0.29 ^efgh^	11.44 ± 2.82 ^mno^	40.96 ± 13.02 ^ijklm^	264.90 ± 22.77 ^defgh^	7.95 ± 1.00 ^efg^
	Baking	134.59 ± 10.94 ^f^	5.27 ± 0.34 ^i^	14.27 ± 2.39 ^st^	0.40 ± 0.11 ^h^	8.33 ± 2.52 ^o^	58.78 ± 13.28 ^f^	277.62 ± 40.00 ^defg^	6.94 ± 0.73 ^ij^
	Frying	99.71 ± 9.89 ^ij^	6.72 ± 1.43 ^de^	30.13 ± 5.67 ^pq^	0.56 ± 0.17 ^gh^	10.85 ± 2.40 ^mno^	44.76 ± 15.75 ^ghijkl^	248.35 ± 38.71 ^fghi^	7.06 ± 1.06 ^hij^
	Microwaving	113.26 ± 10.45 ^h^	7.09 ± 1.36 ^d^	17.64 ± 3.47 ^rst^	1.07 ± 0.14 f ^gh^	26.21 ± 0.30 ^kl^	41.42 ± 12.17 ^hijklm^	292.46 ± 38.48 ^d^	6.61 ± 0.78 ^j^
NAROSPOT 1	Raw	6.18 ± 1.19 ^mno^	0.97 ± 0.19 ^q^	89.57 ± 5.19 ^i^	2.44 ± 0.04 ^e^	105.70 ± 13.07 ^c^	35.07 ± 6.46 ^klm^	151.68 ± 18.75 ^o^	1.87 ± 0.03 ^pq^
	Boiling	17.17 ± 7.23 ^klmno^	5.74 ± 2.77 ^ghi^	71.17 ± 6.73 ^kl^	1.89 ± 0.70 ^ef^	43.59 ± 6.97 ^ghi^	22.22 ± 10.99 ^n^	218.68 ± 45.41 ^ijkl^	3.28 ± 0.71 ^kl^
	Steaming	13.80 ± 3.21 ^klmno^	3.92 ± 1.68 ^j^	65.02 ± 9.11 ^lmn^	1.91 ± 0.55 ^ef^	38.67 ± 1.70 ^ij^	29.75 ± 7.62 ^mn^	169.68 ± 15.62 ^mno^	2.63 ± 1.24 ^lmno^
	Baking	21.55 ± 3.18 ^k^	2.46 ± 0.26 ^nop^	65.18 ± 7.69 ^lm^	1.64 ± 0.46 ^efgh^	51.22 ± 3.44 ^g^	39.61 ± 9.92 ^jklm^	214.84 ± 16.71 ^ijkl^	2.22 ± 0.79 ^opq^
	Frying	5.96 ± 1.18 ^no^	3.46 ± 1.23 ^jkl^	86.52 ± 4.47 ^ij^	1.85 ± 0.65 ^efg^	37.02 ± 4.18 ^ij^	31.45 ± 6.79 ^lmn^	161.29 ± 17.19 ^no^	2.73 ± 0.50 ^klmno^
	Microwaving	19.23 ± 4.33 ^kl^	3.47 ± 1.18 ^jkl^	56.46 ± 5.80 ^n^	2.43 ± 0.06 ^e^	68.63 ± 4.69 ^f^	30.63 ± 7.77 ^mn^	191.79 ± 16.01 ^klmn^	2.91 ± 0.48 ^klmn^
NASPOT 8	Raw	4.95 ± 0.50 ^o^	0.80 ± 0.26 ^q^	172.76 ± 5.55 ^e^	1.63 ± 0.36 ^efgh^	133.74 ± 25.85 ^a^	83.46 ± 13.57 ^cd^	198.68 ± 23.42 ^jklmn^	0.89 ± 0.46 ^r^
	Boiling	11.35 ± 5.09 ^klmno^	5.65 ± 1.11 ^hi^	118.17 ± 4.43 ^h^	1.33 ± 0.19 ^efgh^	42.22 ± 7.87 ^hi^	40.78 ± 7.63 ^ijklm^	224.68 ± 27.31 ^ijk^	3.16 ± 1.36 ^kl^
	Steaming	9.74 ± 0.38 ^klmno^	2.91 ± 0.39 ^lmn^	131.20 ± 5.50 ^g^	1.30 ± 0.12 ^efgh^	38.19 ± 10.19 ^ij^	50.98 ± 18.95 ^fghij^	240.46 ± 24.07 ^ghi^	2.71 ± 0.64 ^klmno^
	Baking	12.02 ± 3.59 ^klmno^	2.05 ± 0.86 ^p^	132.73 ± 6.34 ^g^	0.94 ± 0.16 ^fgh^	39.81 ± 10.24 ^hi^	83.48 ± 13.57 ^cd^	200.90 ± 31.54 ^jklm^	3.00 ± 1.49 ^klm^
	Frying	5.67 ± 1.43 ^o^	2.86 ± 0.08 ^lmno^	152.93 ± 5.41 ^f^	1.02 ± 0.84 ^fgh^	47.75 ± 11.60 ^gh^	73.33 ± 13.81 ^de^	183.01 ± 22.71 ^lmno^	2.69 ± 0.67 ^lmno^
	Microwaving	8.40 ± 1.15 ^lmno^	2.26 ± 0.60 ^op^	133.45 ± 5.00 ^g^	1.49 ± 0.34 ^efgh^	91.91 ± 13.88 ^d^	60.21 ± 6.72 ^ef^	226.79 ± 24.29 ^ijk^	2.24 ± 0.42 ^nopq^
NASPOT 13 O	Raw	5.69 ± 1.82 ^o^	1.09 ± 0.33 ^q^	269.81 ± 18.49 ^b^	0.99 ± 0.25 ^fgh^	124.76 ± 32.26 ^b^	80.86 ± 15.25 ^cd^	232.29 ± 26.52 ^hij^	0.98 ± 0.46 ^r^
	Boiling	18.45 ± 6.31 ^klm^	3.64 ± 0.36 ^jk^	203.93 ± 19.08 ^d^	0.89 ± 0.28 ^fgh^	60.51 ± 11.80 ^f^	48.32 ± 10.30 ^fghijk^	250.73 ± 31.24 ^efghi^	2.88 ± 0.58 ^klmno^
	Steaming	18.09 ± 6.31 ^klmn^	3.23 ± 0.19 ^klm^	267.64 ± 19.32 ^b^	0.83 ± 0.29 ^fgh^	42.83 ± 12.72 ^hi^	54.76 ± 18.36 ^fgh^	249.57 ± 25.99 ^fghi^	3.37 ± 0.53 ^k^
	Baking	18.86 ± 1.45 ^kl^	3.08 ± 0.70 ^klmn^	168.52 ± 16.20 ^e^	0.59 ± 0.14 ^gh^	36.60 ± 5.09 ^ij^	90.06 ± 10.72 ^c^	278.73 ± 25.27 ^def^	2.43 ± 0.40 ^mnop^
	Frying	7.48 ± 2.97 ^lmno^	2.80 ± 0.20 ^mno^	304.74 ± 16.56 ^a^	0.55 ± 0.15 ^h^	40.04 ± 12.47 ^hi^	59.00 ± 12.18 ^f^	201.18 ± 25.12 ^jklm^	2.40 ± 0.69 ^mnop^
	Microwaving	15.12 ± 4.57 ^klmno^	2.95 ± 0.32 ^lmn^	241.29 ± 18.25 ^c^	0.72 ± 0.22 ^fgh^	83.16 ± 19.76 ^e^	53.22 ± 17.65 ^fghi^	242.35 ± 37.90 ^fghi^	2.64 ± 0.53 ^lmno^
PF-167	Raw	110.91 ± 16.67 ^hi^	1.19 ± 0.10 ^q^	78.70 ± 2.13 ^jk^	15.29 ± 2.80 ^b^	89.34 ± 11.42 ^de^	113.65 ± 23.12 ^b^	215.12 ± 25.95 ^ijkl^	1.68 ± 0.55 ^q^
	Boiling	158.90 ± 9.56 ^de^	9.21 ± 0.25 ^a^	64.75 ± 7.12 ^lmn^	17.13 ± 3.14 ^a^	18.17 ± 8.39 ^lmn^	48.32 ± 18.21 ^fghijk^	416.45 ± 33.47 ^bc^	10.95 ± 1.59 ^b^
	Steaming	137.09 ± 19.46 ^f^	8.46 ± 1.12 ^bc^	57.38 ± 2.61 ^mn^	16.67 ± 3.05 ^a^	12.68 ± 3.69 ^mno^	55.69 ± 13.46 ^fg^	404.68 ± 22.92 ^bc^	9.72 ± 1.09 ^c^
	Baking	180.89 ± 15.60 ^ab^	9.10 ± 0.67 ^ab^	47.40 ± 4.99 ^o^	12.23 ± 2.24 ^d^	14.21 ± 2.41 ^mno^	126.60 ± 24.37 ^b^	399.68 ± 45.18 ^c^	9.17 ± 0.92 ^cd^
	Frying	154.58 ± 19.09 ^e^	8.40 ± 0.67 ^c^	69.47 ± 3.55 ^l^	13.92 ± 2.55 ^c^	17.28 ± 6.72 ^mn^	81.08 ± 12.61 ^cd^	415.29 ± 47.54 ^bc^	8.52 ± 0.49 ^de^
	Microwaving	155.37 ± 14.90 ^e^	8.31 ± 1.24 ^c^	44.61 ± 0.85 ^o^	17.43 ± 3.19 ^a^	31.38 ± 4.35 ^jk^	56.21 ± 13.05 ^fg^	422.12 ± 32.29 ^bc^	12.23 ± 1.24 ^a^
*p*-value		<0.001	<0.001	<0.001	<0.001	<0.001	<0.001	<0.001	<0.001

All values are on a dry weight basis. Values are means ± SD of three independent biological replicates (*n* = 3). Means in the same column with different superscripts are significantly different (*p* < 0.001). TPC = total phenolic compounds; GAE = gallic acid equivalent; TFC = total flavonoid content; QE = quercetin equivalent; TCC = total carotenoid content; TMAC = total monomeric anthocyanin content; VC = vitamin C; AAE = ascorbic acid equivalent; TAL = total alkaloid content; CE = catechin equivalent; TSC = total saponin content; AE = aescin equivalent; TTC = total tannin content; TA = tannic acid.

**Table 3:** Bioactive compound content of sweetpotato storage roots as influenced by peeling.

Peel Condition	TPC (mg GAE/g)	TFC (mg QE/g)	TCC (µg/g)	TMAC (mg/g)	VC (µg AAE/g)	TAL (µg CE/g)	TSC (mg AE/g)	TTC (mg TA/g)
WP	91.18 ± 48.70 ^a^	5.45 ± 2.83 ^a^	98.33 ± 46.15 ^a^	4.46 ± 3.74 ^a^	56.18 ± 25.24 ^a^	90.21 ± 54.36 ^a^	301.60 ± 110.22 ^a^	5.49 ± 3.25 ^a^
WTP	62.90 ± 30.83 ^b^	3.65 ± 2.50 ^b^	85.71 ± 38.74 ^b^	2.58 ± 1.33 ^b^	33.79 ± 19.87 ^b^	35.41 ± 16.41 ^b^	244.96 ± 94.26 ^b^	4.25 ± 2.07 ^b^
*p*-value	<0.001	<0.001	<0.001	<0.001	<0.001	<0.001	<0.001	<0.001

All values are on a dry weight basis. Values are means ± SD of three independent biological replicates (*n* = 3). Means in the same column with different superscripts are significantly different (*p* < 0.001). WP = with peel; WTP = without peel; TPC = total phenolic compounds; GAE = gallic acid equivalent; TFC = total flavonoid content; QE = quercetin equivalent; TCC = total carotenoid content; TMAC = total monomeric anthocyanin content; VC = vitamin C; AAE = ascorbic acid equivalent; TAL = total alkaloid content; CE = catechin equivalent; TSC = total saponins content; AE = aescin equivalent; TTC = total tannin content; TA = tannic acid.

## Data Availability

All of the data is contained within the article.

## Supplementary Materials

The following supporting information can be downloaded at: https://www.mdpi.com/article/10.3390/antiox11101867/s1: Table S1: Bioactive compounds content of sweetpotato storage roots as influenced by cooking method and peel condition; Table S2: Bioactive compound content of sweetpotato storage roots as influenced by genotype and peel condition; Figure S1: Antioxidant activities of sweetpotato storage roots as influenced by cooking method and peel condition; Figure S2: Antioxidant activities of sweetpotato storage roots as influenced by genotype and peel condition.
