# Supplementary material for: Household Processing Methods and Their Impact on Bioactive Compounds and Antioxidant Activities of Sweetpotato Genotypes of Varying Storage Root Flesh Colours
Source: Antioxidants (Basel). 2022 Sep 21;11(10):1867. doi: 10.3390/antiox11101867 (PMC9598835; doi:10.3390/antiox11101867)
Supplement: Supplementary file 1 [file antioxidants-11-01867-s001.zip › antioxidants-1889864-supplementary.pdf]

Supplementary Data

Table S1. Bioactive compounds content of sweetpotato storage roots as influenced by cooking method and peel condition

**Cooking method**	**Peel condition**	**TPC (mg GAE/g)**	**TFC (mg QE/g)**	**TCC (µg/g)**	**TMAC (mg/g)**	**VC (µg AAE/g)**	**TAL (µg CE/g)**	**TSC (mg AE/g)**	**TTC (mg TA/g)**
Raw	WP	63.17 ± 15.62[ef]	1.28 ± 0.22[e]	119.27 ± 53.92[a]	4.83 ± 1.59[a]	124.31 ± 47.88[a]	127.35 ± 53.43[b]	228.51 ± 93.61[g]	2.27 ± 1.11[f]
	WTP	39.59 ± 12.32[g]	0.82 ± 0.18[f]	104.26 ± 52.65[c]	2.64 ± 1.31[de]	85.44 ± 30.36[b]	46.47 ± 15.10[f]	165.53 ± 53.53[h]	1.05 ± 0.61[g]
Boiling	WP	105.98 ± 18.22[a]	7.48 ± 1.71[a]	85.02 ± 49.66[e]	4.87 ± 2.44[a]	38.37 ± 10.35[d]	60.52 ± 19.43[e]	303.44 ± 75.17[bc]	6.61 ± 3.30[a]
	WTP	70.75 ± 15.96[d]	5.27 ± 2.43[c]	78.42 ± 44.91[f]	2.84 ± 1.00[cd]	25.32 ± 7.24[f]	21.10 ± 6.18[i]	232.40 ± 80.69[fg]	5.22 ± 3.13[cd]
Steaming	WP	96.65 ± 18.99[b]	6.36 ± 2.12[b]	103.23 ± 67.23[c]	4.75 ± 1.27[a]	32.13 ± 8.99[e]	69.94 ± 21.19[d]	331.14 ± 92.99[a]	6.38 ± 3.09[a]
	WTP	61.27 ± 19.50[f]	3.93 ± 2.12[d]	83.98 ± 41.52[e]	2.77 ± 1.84[cde]	18.61 ± 7.67[g]	26.73 ± 3.49[hi]	252.85 ± 83.87[ef]	4.96 ± 2.91[d]
Baking	WP	98.36 ± 17.08[b]	5.22 ± 2.59[c]	78.59 ± 44.30[f]	3.40 ± 1.67[bc]	34.16 ± 11.00[de]	137.34 ± 52.51[a]	329.01 ± 75.52[a]	5.83 ± 2.83[b]
	WTP	68.71 ± 11.81[de]	3.99 ± 2.53[d]	67.93 ± 36.64[g]	2.03 ± 0.29[e]	19.39 ± 5.17[g]	48.09 ± 18.38[f]	291.97 ± 93.61[cd]	4.56 ± 2.92[e]
Frying	WP	88.01 ± 11.70[c]	6.20 ± 2.18[b]	112.15 ± 83.71[b]	3.83 ± 1.33[b]	36.15 ± 7.85[de]	78.22 ± 29.70[c]	296.20 ± 86.55[c]	5.51 ± 2.59[bc]
	WTP	70.49 ± 11.02[d]	3.93 ± 2.43[d]	102.52 ± 62.55[c]	2.29 ± 0.88[de]	19.32 ± 7.26[g]	37.13 ± 13.35[g]	253.49 ± 87.02[ef]	4.49 ± 2.56[e]
Microwaving	WP	94.90 ± 10.24[bc]	6.16 ± 2.58[b]	91.75 ± 55.81[d]	5.07 ± 2.55[a]	71.96 ± 24.63[c]	67.90 ± 19.44[de]	321.29 ± 98.44[ab]	6.35 ± 4.02[a]
	WTP	66.63 ± 13.13[def]	3.96 ± 2.25[d]	77.18 ± 30.43[f]	2.89 ± 1.11[cd]	34.68 ± 11.17[de]	32.96 ± 10.90[gh]	273.55 ± 95.41[de]	5.22 ± 3.48[cd]
P-value		0.003	<0.001	0.008	0.542	<0.001	<0.001	0.050	0.705

All values are on dry weight basis. Values are means ± SD of three independent biological replicates (n=3). Means in the same column with different superscripts are significantly different ($P < 0.05$). WP=with peel; WTP=without peel; TPC=total phenolic compounds; GAE=gallic acid equivalent; TFC=total flavonoids content; QE=quercetin equivalent; TCC=total carotenoids content; TMAC=total monomeric anthocyanin content; VC=vitamin C; AAE=ascorbic acid equivalent; TAL=total alkaloids content; CE=catechin equivalent; TSC=total saponins content; AE=aescin equivalent; TTC=total tannins content; TA=tannic acid.

Supplementary Data

Table S2. Bioactive compounds content of sweetpotato storage roots as influenced by genotype and peel condition

Genotype	Peel condition	TPC (mg GAE/g)	TFC (mg QE/g)	TCC (µg/g)	TMAC (mg/g)	VC (µg AAE/g)	TAL (µg CE/g)	TSC (mg AE/g)	TTC (mg TA/g)
'Ssetyabule'	WP	185.30 ± 33.95[a]	6.45 ± 2.35[c]	14.93 ± 5.04[ij]	1.27 ± 0.46[ef]	20.04 ± 11.80[f]	132.99 ± 80.41[a]	440.47 ± 78.87[a]	7.35 ± 1.89[c]
	WTP	137.09 ± 40.24[c]	3.92 ± 1.47[f]	11.64 ± 5.74[j]	0.02 ± 0.01[h]	12.59 ± 10.14[g]	46.79 ± 21.47[e]	335.18 ± 102.74[c]	5.69 ± 2.09[d]
NASPOT 11	WP	136.87 ± 27.22[c]	6.61 ± 2.78[bc]	27.00 ± 9.89[h]	1.83 ± 0.79[de]	43.42 ± 18.01[c]	59.69 ± 14.36[d]	263.21 ± 75.61[d]	6.99 ± 2.32[c]
	WTP	92.76 ± 18.22[e]	4.96 ± 2.18[d]	19.59 ± 6.25[i]	0.06 ± 0.03[h]	27.31 ± 11.51[e]	31.38 ± 8.58f[g]	203.62 ± 67.37[g]	5.97 ± 2.29[d]
NAROSPOT 1	WP	18.61 ± 6.32[fg]	4.53 ± 2.38[e]	77.43 ± 10.48[e]	3.58 ± 0.71[c]	71.02 ± 31.28[b]	38.21 ± 6.32[f]	198.31 ± 35.26[g]	3.25 ± 0.61[e]
	WTP	9.35 ± 6.68[hi]	2.14 ± 0.83[h]	67.21 ± 16.71[f]	0.47 ± 0.13[gh]	43.92 ± 21.89[c]	24.70 ± 6.95[g]	171.01 ± 33.07[h]	1.96 ± 0.66[g]
NASPOT 8	WP	12.97 ± 3.06[gh]	3.50 ± 1.75[g]	147.75 ± 25.11[c]	2.28 ± 0.56[d]	84.92 ± 47.93[a]	81.79 ± 17.21[c]	236.31 ± 53.21[ef]	3.17 ± 1.13[ef]
	WTP	4.41 ± 3.12[i]	2.01 ± 1.41[h]	132.66 ± 18.24[d]	0.30 ± 0.11[gh]	46.29 ± 27.47[c]	48.95 ± 21.93[e]	188.53 ± 25.18[gh]	1.73 ± 0.62[g]
NASPOT 13	WP	20.19 ± 8.12[f]	3.66 ± 1.19[fg]	256.51 ± 48.28[a]	0.95 ± 0.21[fg]	81.52 ± 39.70[a]	99.19 ± 30.50[b]	256.68 ± 28.30[de]	2.83 ± 0.82[f]
	WTP	7.70 ± 3.81[hi]	1.93 ± 0.67[h]	228.80 ± 47.85[b]	0.57 ± 0.14f[gh]	47.78 ± 25.50[c]	29.55 ± 5.14[g]	228.27 ± 46.13[f]	2.07 ± 0.82[g]
PF-167	WP	173.14 ± 29.81[b]	7.96 ± 3.10[a]	66.38 ± 12.26[f]	16.83 ± 2.60[a]	36.16 ± 27.58[d]	129.42 ± 55.53[a]	414.60 ± 78.73[b]	9.35 ± 3.87[a]
	WTP	126.10 ± 19.49[d]	6.93 ± 2.79[b]	54.39 ± 13.88[g]	14.06 ± 3.30[b]	24.86 ± 18.67[e]	31.10 ± 8.90f[g]	343.18 ± 82.93[c]	8.07 ± 3.19[b]
P-value		<0.001	<0.001	<0.001	<0.001	<0.001	<0.001	<0.001	0.030

All values are on dry weight basis. Values are means ± SD of three independent biological replicates (n=3). Means in the same column with different superscripts are significantly different ($P < 0.05$). WP=with peel; WTP=without peel; TPC=total phenolic compounds; GAE=gallic acid equivalent; TFC=total flavonoids content; QE=quercetin equivalent; TCC=total carotenoids content; TMAC=total monomeric anthocyanin content; VC=vitamin C; AAE=ascorbic acid equivalent; TAL=total alkaloids content; CE=catechin equivalent; TSC=total saponins content; AE=aescin equivalent; TTC=total tannins content; TA=tannic acid.

Figure S1. Antioxidant activities (ABTS and FRAP) of sweetpotato storage roots as influenced by cooking method and peel condition

1A

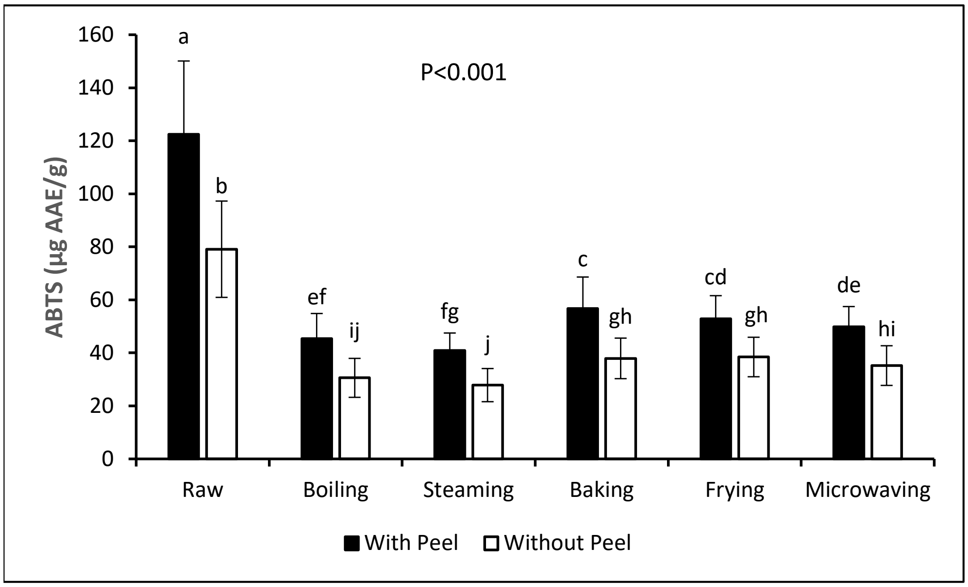

1B

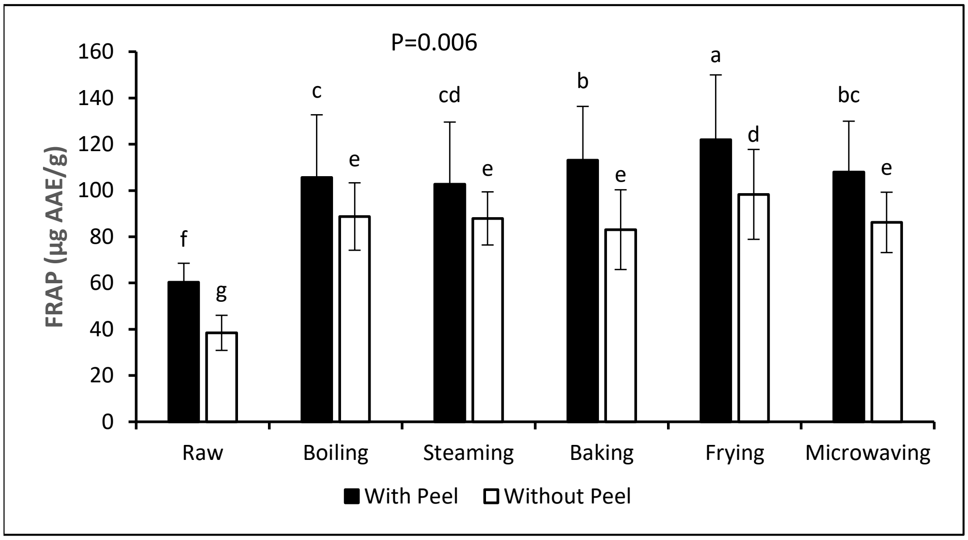

Figure S1. Changes in antioxidant activities by ABTS assay (A) and FRAP assay (B) of sweetpotato genotypes as influenced by cooking method and peel condition. Each bar represents the mean of three independent biological replicates (n=3) with error bar showing the standard deviation. Bars with different letters on top are significantly different at $P < 0.05$.

Figure S2. Antioxidant activities (ABTS and FRAP) of sweetpotato storage roots as influenced by genotype and peel condition

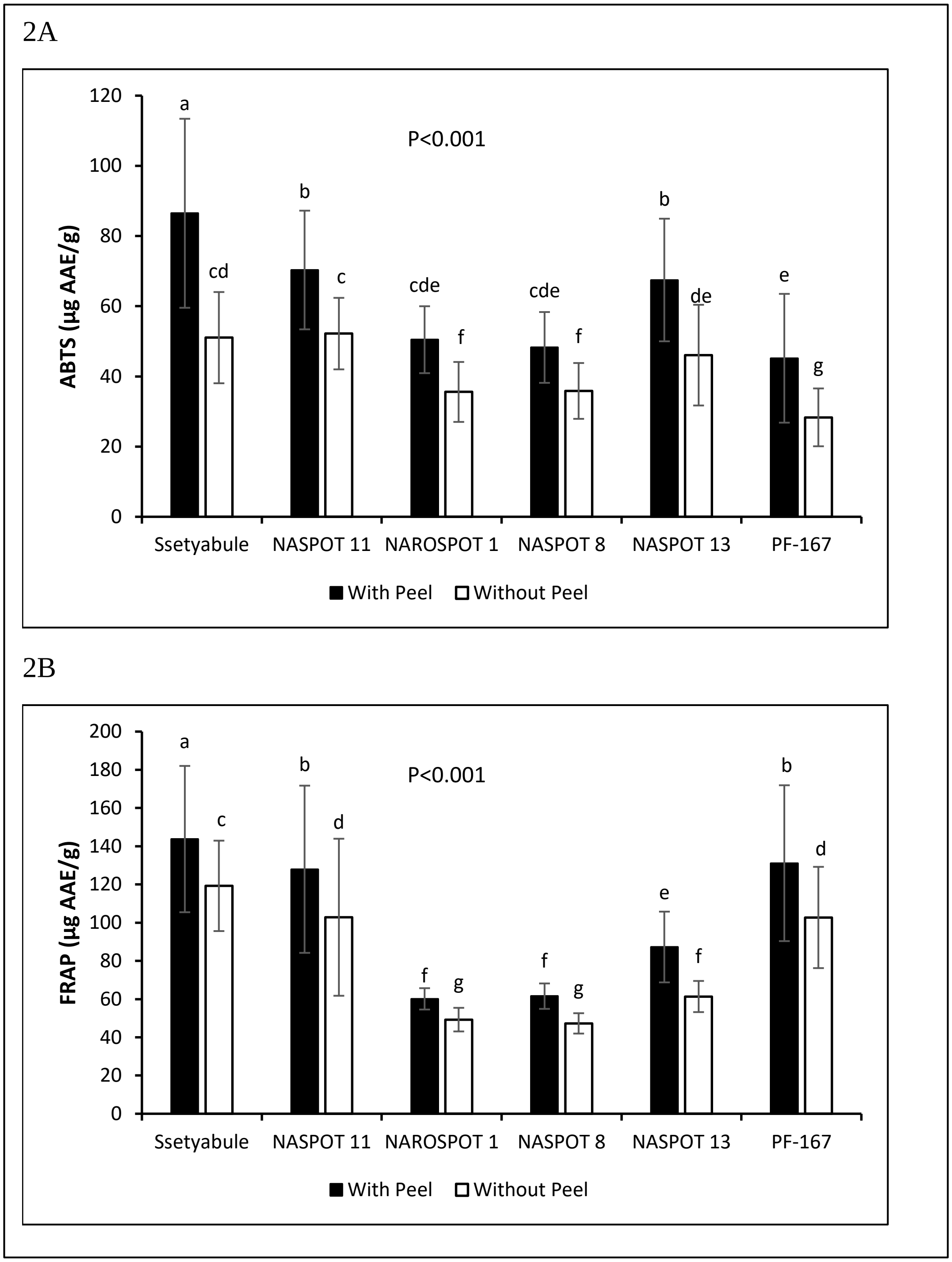

Figure S2. Changes in antioxidant activities by ABTS assay (2A) and FRAP assay (2B) of sweetpotato genotypes as influenced by genotype and peel condition. Each bar represents the mean of three independent biological replicates (n=3) with error bar showing the standard deviation. Bars with different letters on top are significantly different at $P < 0.001$.
